# Trajectories of Dynamic Risk Factors During Forensic Treatment: Growth Trajectory of Clinical Risk Factors in a Sample of Dutch Forensic Patients

**DOI:** 10.1177/0306624X20909219

**Published:** 2020-03-02

**Authors:** Robin Van der Linde, Stefan Bogaerts, Carlo Garofalo, Eric Blaauw, Elien De Caluwé, Eva Billen, Marinus Spreen

**Affiliations:** 1 Tilburg University, the Netherlands; 2 Fivoor Science and Treatment Innovation, Rotterdam, the Netherlands; 3 Verslavingszorg Noord Nederland, Groningen, the Netherlands; 4 Hanze University of Applied Sciences, Groningen, the Netherlands; 5 Stenden University of Applied Sciences, Leeuwarden, the Netherlands

**Keywords:** forensic psychiatry, HKT-R, risk factors, latent growth analysis

## Abstract

In this study, growth trajectories (from admission until unconditional release) of crime-related dynamic risk factors were investigated in a sample of Dutch forensic patients (*N* = 317), using latent growth curve modeling. After testing the unconditional model, three predictors were added: first-time offender versus recidivist, age, and treatment duration. Postanalyses were chi-square difference tests, *t* tests, and analyses of variance (ANOVAs) to assess differences in trajectories. Overall, on scale level, a decrease of risk factors over time was found. The predictors showed no significant slope differences although age and treatment duration differed significantly at some time points. The oldest age group performed worse, especially at later time points. Treatment duration effects were found at the second time point. Our results that forensic patients show a decrease in crime-related risk factors may indicate that treatment is effective. This study also found differences in growth rates, indicating the effect of individual differences

## Introduction

In many countries, forensic psychiatric patients are admitted to secure forensic institutions because they have committed a violent crime caused by severe mental disorders. The main goal of staying in low, medium, or high secure services is to receive treatment for offense-related disorders or risk factors, such as impulsivity and hostility, and to allow these patients to reintegrate into society on condition that there has been a change in the severity of risk factors that justify a return to society (Bogaerts et al., 2018). Studies aimed at obtaining longitudinal insights into the changeability of clinical reversible risk factors are very scarce. Within the broader context of treatment outcomes, longitudinal research on the changeability of problematic criminally oriented behavior characterized by severe offense-related dynamic risk factors is rarely done because the length of stay in forensic settings is often very long what makes longitudinal follow-up very time-intensive and time consuming. Furthermore, because of the specificity of high-risk forensic psychiatric patients staying in high secure services, it’s difficult to meet the assumption of sufficient power.

The Dutch forensic psychiatric context is quite exceptional compared with other countries. All patients in this study were sentenced with a TBS order (“terbeschikkingsstelling”; meaning involuntary admission by order of the state), which is a Dutch criminal law measure. A TBS order means that patients are not responsible for their behavior, which can vary from fully accountable to completely irresponsible (full responsibility, slightly diminished responsibility, diminished responsibility, severely diminished responsibility, and total absence of responsibility). Prior to compulsory treatment, a prison sentence is first imposed by the court (De Ruiter & Trestman, 2007). A TBS order is a mandatory admission to a high-security forensic psychiatric center (FPC) for mandatory treatment by order of the state because of mental disorder that is related to the committed crime (De Ruiter & Trestman, 2007). The offense committed must have a criminal threat of at least 4 years. The goal of a TBS order is the protection of society and the rehabilitation of patients into society.

Numerous studies have shown that the presence of risk factors and a lack of protective factors have moderate to strong associations with recidivism (Beech et al., 2002; Bogaerts et al., 2018; Mulder et al., 2010). However, only a few studies show whether risk factors can actually be reduced and reinforced longitudinally during a stay in high security institutions, such as FPCs (Van der Veeken et al., 2018). Most studies only use a cross-sectional research design and examine the predictive contribution of these risk factors in the prediction and occurrence of recidivism, but do not address the course of these risk and protective factors over time during treatment.

For many years, the forensic domain has focused on risk and protective factors, which are empirically related to the risk of recidivism. To construct fitting assessment and treatment, the risk-need-responsivity (RNR) model is developed (Andrews & Bonta, 2006; Andrews et al., 1990). This theoretical model is one of the leading models and has been developed within a general personality and cognitive social learning theory of criminal behavior. The RNR model employs three different principles. The risk principle indicates that recidivism can be reduced if the level and intensity of treatment are proportional to an offender’s risk of reoffending. The need principle emphasizes the criminogenic needs that should be the objective of treatment interventions. These criminogenic needs, such as procriminal attitudes and antisocial cognitions, are risk factors directly associated with recidivism, which can be influenced by treatment interventions (Andrews & Bonta, 2006). The responsivity principle states how treatment should be provided by adapting the treatment to the characteristics, learning styles, and abilities of the patient (Bonta & Andrews, 2007; Van der Veeken et al., 2018). According to Andrews and Bonta (2006), an average recidivism reduction of 17% can be established between treated and nontreated offenders when the RNR model is included in a treatment program.

## Criminogenic Needs as Target of Treatment

Following the RNR model, criminogenic needs are operationalized as the central eight risk/needs factors, which are subdivided into the big four and the moderate four (Andrews & Bonta, 2006). The big four (history of antisocial personality patterns, antisocial behavior, antisocial cognitions, and antisocial peers) are strongly predictive of criminal behavior and reoffending, while the moderate four (poor school and work performance, substance abuse, poor family and marital relationships, and a lack of prosocial recreational activities) have an indirect effect on reoffending (Andrews & Dowden, 2006).

In this study, the focus is on five of the central eight factors, as they can be changed during treatment in a FPC, namely antisocial personality patterns, antisocial cognitions, antisocial peers, poor school and work performance, and substance abuse. Antisocial personality is characterized by impulsive, irresponsible, and hostile behavior. Antisocial personality disordered individuals typically will feel hostility towards the world, lack adequate coping skills, and violate agreements that are made in the FPC (Hare et al., 1991). Antisocial cognitions refer to attitudes, believes, and thoughts that support crime, such as a lack of problem insight and irresponsibility for the committed offense (Walters & DeLisi, 2013). Antisocial peers are part of the antisocial network of the patient and perform behavior such as drug use or stealing that influence the deviant behavior of the patient (Kaplan et al., 1987). Finally, also psychotic symptoms and a lack of self-reliance have been investigated in the current study because these factors are indicators of reoffending. Having psychotic symptoms can evoke antisocial cognitions and attitudes and self-reliance is often a prodromal sign of a psychotic episode (Skeem et al., 2014). These five central factors, psychotic symptoms and self-reliance can be measured by clinical risk assessment instruments, such as the Historical, Clinical, and Future–Revised (HKT-R [*Historisch Klinisch Toekomst–Revised*]; Spreen et al., 2013).

## Reduction of Risk Factors and Growth Trajectories

Longitudinal change of previously mentioned clinical risk factors can be assessed by investigating forensic patients’ risk scores over time. Although the golden standard to measure risk level changes is pre–post measurement, forensic patients show complex behavior, and have relatively high drop-out, which makes a randomized control trial hardly feasible (Woicik et al., 2017). Therefore, routinely assessing levels of risk factors at multiple time points is a strong alternative (Ellwood, 1998; Van der Veeken et al., 2018).

Various risk assessment instruments have been developed, such as the worldwide used Historical Clinical Risk Management-20 Version 3 and the Dutch HKT-R, which is mandatory in the Netherlands to measure change in recidivism risk over time. In this study, we assess longitudinal trajectories of dynamic clinical risk factors in offenders who received a TBS order following their committed crime. Confinement TBS order is stopped only when the patient’s risk is sufficiently reduced (Van Nieuwenhuizen et al., 2011). Treatment options in forensic psychiatry are, for example, cognitive behavioral therapy, schema focus therapy, psychomotoric therapy, music therapy, psychopharmaceutical therapy, and a combination of therapies.

Forensic patients with a TBS order are very heterogeneous in terms of psychopathology, risk and protective factors, and type of offense committed. Because of this heterogeneity, it is necessary to investigate these differences to provide better treatment outcomes. Taking into account individual patient differences can refine treatment decisions to obtain the largest guarantee of relapse prevention. In this study, three differentiating characteristics were investigated. The first is whether patients are recidivists or first-time offenders at the time of the index offense for which the TBS order was imposed. The second aspect is the patients’ age when admitted to the FPCs, and the third refers to the duration of their stay or treatment in the FPCs. Because of the complexity of psychiatric disorders and the tenacity of clinical risk factors to change, a decrease in clinical risk factors is not as salient as expected (Van der Veeken et al., 2018). Clinical studies show mixed results regarding the severity decrease of clinical risk factors. For example, De Jonge et al. (2009), studied 984 HKT-30 scores (the predecessor of the HKT-R) and found a decrease of clinical risk factors in a Dutch forensic population in three different FPCs. However, Van der Veeken et al. (2018) found no significant progress over time in 240 patients (total group) from two Dutch FPCs based on their scores on problematic behavior, protective behavior, and resocialization skills (Schuringa et al., 2018). However, she did find a significant decrease in problematic behavior in patients scoring very high at problematic behavior at admission and an improvement of protective and resocialisation behavior in patients scoring problematic on both factors at admission. Because the changeability of dynamic risk factors has rarely been investigated thus far, this study is the first to investigate long-term trajectories with regard to the change of dynamic clinical risk factors among all TBS patients who were unconditionally released in the Netherlands between 2004 and 2008.

### First-Time Offenders or Recidivists

The first differentiating characteristic investigated in this study is whether patients are first-time offenders or recidivists before they were sentenced to the TBS order. Offenders receiving a TBS order are usually first sent to prison before entering a FPC. Especially the effect of imprisonment on first-time offenders compared with recidivists has been investigated (Clear, 1996; Doob et al., 2014; Durlauf & Nagin, 2011). Incarceration effects are mixed, on one hand, imprisonment can have a deterrent impact on prisoners, that is, crime is prevented through the experience of an actual sanction. On the other hand, crime-enhancing effects of imprisonment are found (Clear, 1996; Vieraitis et al., 2007), that is, imprisonment on its own has a criminogenic effect. Incarceration also affects cognitive, emotional, and volitional aspects of personality (Flórez, 2009; Okasha, 2004). Offenders in prison experience more social maladjustment, more substance abuse, and an increase in psychotic symptoms (Fazel et al., 2016). Because of incarceration effects and the adverse effects of imprisonment on mental health, it can be expected that there are differences between patients who have been sentenced to prison previously, compared with patients who have only been convicted once. Therefore, this study investigated whether the growth trajectories of the clinical risk scale for first-time offenders differed from those of recidivists during their stay in the FPCs.

### Age of Admission

The second differentiating characteristic is age of admission. Wilpert et al. (2018) conducted a study on the central eight factors as predictors of recidivism in different age groups of sex offenders. Results showed that the factors of the central eight differed between age groups. The youngest age group (<18 years) demonstrated the most problems in several areas of the central eight, such as school/work and antisocial cognitions, whereas the oldest age group (more than 55 years), showed the least problems in these eight factors. However, development of these risk and protective factors were not investigated in this study, resulting in the recommendation to a longitudinal design in which developmental trajectories of risk factors can be studied (Wilpert et al., 2018). Following this recommendation, the current study investigated whether the growth trajectory of the clinical risk scale is different for offenders with a different age.

### Length of Stay

There are large differences in length of stay or treatment in Dutch FPCs. Some patients stay for a period of less than 5 years, whereas other patients remain in the FPCs for a period of more than 10 years, with the average length of stay in the Netherlands being 9.8 years (Nagtegaal et al., 2011). A study conducted on the length of stay among 70 females in medium secure settings showed that patients who progressed through medium secure care faster, had greater engagement in therapy, which resulted in a lower level of risk behavior (Long & Dolley, 2011). Conversely, a study on the association between duration of addiction treatment and improvements in drug use found that treatment duration had a positive relationship with primary drug use improvement, and that the improvement for long-term residential clients was the greatest with longer treatment duration (Zhang et al., 2003). These findings show that results regarding the effect of treatment duration are mixed. Therefore, the current study examined whether there is an association between the duration of treatment and the growth curves of the dynamic clinical risk scale of the HKT-R.

## Aim of the Study

The goal of the present study is to investigate changes in the clinical risk scale consisting of clinical risk factors over time in FPCs. The clinical risk scale consisted of 11 clinical risk factors of the 14 clinical factors of the HKT-R. The period to which the measurement was related, was the moment of the first judicial psychiatric assessment until the moment of unconditional release. All patients released from all FPCs in the Netherlands between 2004 and 2008 were included in the study. To assess a possible reduction in the clinical risk scale, a latent growth modeling analysis was performed. Due to expected treatment effects, we expected growth trajectories that show a reduction on the clinical scale (sum of the 11 clinical risk factors). Because there is substantial variation in the literature regarding the three differentiating characteristics of interest, we do not formulate specific hypotheses. Nevertheless, exploration of these characteristics is relevant as different patient groups may show different growth trajectories on the clinical risk scale. Thus, it is investigated whether there are differences between first-time offenders and recidivists, between different age groups, and between different treatment durations.

## Method

### Procedure

In 2009, the Dutch Ministry of Security and Justice commissioned three FPCs (FPC Kijvelanden, FPC Dr. S. van Mesdag, and Forensic Pyschiatric Clinic [FPK] Woenselsepoort), and Tilburg University to revise the HKT-30, which led to the current HKT-R. For scoring the patient files, an experimental version of the HKT-R was developed, which consisted of the 33 HKT-R items, supplemented with a number of items that were marked mainly by the clinical field as important items to be included in a follow-up version of the HKT-30. All patients of the 12 FPCs in the Netherlands, who were unconditionally released between 2004 and 2008 were included in the study (*N* = 347). The Ministry of Security and Justice gave permission to investigate the individual electronic patient files, which were stored at two locations in a secure research environment, namely The Dutch Justice Department in The Hague and the FPC Dr. S. van Mesdag in Groningen. Electronic patient files contains systematic patient information, such as criminal history, risk and protective factors based on risk assessment instruments, such as the HKT-R, diagnoses according to *Diagnostic and Statistical Manual of Mental Disorders* (4th ed., text rev.; *DSM-IV-TR*; American Psychiatric Association [APA], 2000) and *Diagnostic and Statistical Manual of Mental Disorders* (5th ed.; *DSM-5*; APA, 2013), demographics, such as age and marital status, medication, treatment information, such as type of treatment, and treatment progress information in term of decrease, increase or stagnation of risk factors. These files are managed by an application manager; researchers are not allowed to have access to these files but receive patient information in an anonymous and encrypted format. Under supervision of two of the four developers of the HKT-R, Dr. Spreen, and Dr. Brand, 10 psychology students were trained in scoring the patient files. Before the students had access to the files, they signed a confidentiality agreement. To ensure scoring integrity, 60 randomly selected cases (*n* = 12 files from each of the 5 years between 2004 and 2008) were scored by two independent raters (the last author and a researcher not involved in this publication) to calculate the interrater reliability. At the domain level, excellent interrater reliability was established for both the historical (intraclass correlation [ICC] = .80) and the Clinical domain (ICC = .85). The interrater reliability of the future domain (ICC = .42) was reasonable, which was mainly due to a lack of distribution in the individual *T*-indicators (restriction of range; Bogaerts et al., 2018; Spreen et al., 2013).

Changes in risk scores were measured retrospectively at five time points. The first assessment of the risk scores took place at the time of the juridical psychiatric observation (performed by a psychiatrist and psychologist) during the police investigation, the time of judicial assessment. Based on the expert’s report, all patients received a TBS order. The second measurement took place after the first 12 months of the stay in the FPCs. The third measurement was scored before the first unguided leave, which means that patients can stay outside the institution for, for example, half a day without supervision. The fourth measurement was before the patients went on conditional leave. During conditional leave, patients live outside the secured zone of the FPC but are still supervised by the FPC. The fifth and last measurement was conducted before the patients were unconditionally released, meaning that they are no longer supervised by correctional services.

### Participants

The original sample of this study (*N =* 347) consisted of all Dutch forensic psychiatric patients who were discharged between 2004 and 2008 from any of the 12 Dutch FPCs. Of all 347 patients, 317 were male (91.4%), and 30 patients were female (8.6%) (Spreen et al., 2013). Female participants were excluded in this study, because the total number of female participants was too small to be included in the analyses (*N* = 30), leaving a total of 317 participants. Patients were of varying nationalities, including Dutch, Moroccan, and Turkish patients. Of these 317 forensic patients, 35 (11%) patients were first-time offenders, which means that they had never committed a criminal offense before the index offense, and 282 (89%) patients had already committed one or more offenses before being convicted to a TBS order. The mean age of the patients at the time of their admission was 31.86 (*SD* = 8.72, range = 17–65) years. The patients were categorized into three age groups: younger than 25 (*n* = 84) years, 25 to 44 (*n* = 205) years, and 45 years and older (*n* = 28). For 138 patients, the treatment duration was unknown. In the period 2004–2008, not many FPCs used electronic patient files but only paper records that gave rise to missing data. In addition, the use of imputation techniques in our research was not justified because data were not missing at random based on preliminary analysis. Therefore, to examine the effect of the length of stay, only 179 patients were included. Of the 179 patients, on average, patients were treated in the clinic for a period of 5.9 years (*SD* = 1.38, range = 3.77–10.99). The patients were divided into three groups based on the duration of stay in the forensic clinic: treatment shorter than 5 years (*n* = 47), treatment of 5 to 7 years (*n* = 100), and treatment of 8 years or more (*n* = 32).

### Measurements

#### Risk assessment

As previously discussed, in this study, the focus is on five of the central eight factors, namely antisocial personality patterns, antisocial cognitions, antisocial peers, poor school and work performance, and substance abuse. History of antisocial behavior is excluded because this static factor is irreversible and only relates to the past. Family and marital relationships, and prosocial recreational activities are excluded because in high-security forensic institutions, contact with intimates and relatives is limited, and prosocial hobbies are not systematically monitored. Antisocial personality refers to impulsivity, current antisocial behavior, antisocial skills, hostility towards others and the world, a lack of adequate problem-solving skills or coping skills, treatment noncompliance, and violation of conditions and agreements. Antisocial cognitions refer to a lack of introspection or problem insight and not taking responsibility for the offense. Antisocial peers or relatives refer to antisocial network members and substance abuse is measured by the general indicator addiction. School or work performance is related to inadequate job skills (Bogaerts et al., 2018). Finally, psychotic symptoms and a lack of self-reliance were added as indicators of reoffending because psychotic symptoms can evoke antisocial cognitions and attitudes and self-reliance is often a prodromal sign of a psychotic episode (Skeem et al., 2014). These five central factors, psychotic symptoms and self-reliance can be measured using clinical scales of risk assessment instruments such as the HKT-R (Spreen et al., 2013).

The HKT-R (Spreen et al., 2013) is the most frequently used risk assessment instrument in the Netherlands. The HKT-R consists of three domains: the Historical domain, the Clinical domain, and the Future domain (Spreen et al., 2013). The items in the domains are scored on a 5-point scale, ranging from 0, *a very low risk at recidivism*, to 4, *a very high risk of recidivism*. The Historical domain contains 12 items and refers to the history of the patients. Items include, for example, *judicial history* and *addiction history.* The Historical domain (not involved in this study) represents static, irreversible, and untreatable factors associated to an offender’s history providing baseline information and the likelihood of future recidivism.

The dynamic risk factors, which are the targets of intervention, consist of clinical and future factors. Dynamic risk factors are susceptible to change and concern psychological and behavioral features of the offender that relate to the risk of reoffending. The Clinical domain (involved in this study) consists of 14 items and regards the behavior of the patient in the FPC 12 months prior to the risk assessment. Clinical factors, such as *antisocial behavior* and *distorted cognitions* relate to the behavior of the patients in treatment in the 12 months prior to the risk assessment evaluation. The Future domain (not involved in this study) consists of seven items, relating to the situations outside the FPC that influence the risk of recidivism. Items include, for example, *employment* and *social network*. Future factors assess the risk of recidivism when patients receive leave modalities or are resigned from the clinic.

In this study, the Clinical domain is investigated, and off the 14 clinical items, 11 items are representing the clinical risk scale, namely *impulsivity, current antisocial behavior, social skills, hostility, coping skills, insight into one’s own problems, responsibility for committed offence, psychotic symptoms, self-reliance, employment skills*, and *protective or risk factors in the network of the patient*. The items *addictive behavior, treatment cooperation*, and *violation of terms and agreements* were excluded due to a high number of missing values. Because the item addictive behavior was excluded from the analyses, the central risk factor substance use could no longer be assessed in this study. Although each clinical item represents a unique risk factor, this study opted to study change in risk scores over time at scale level, thus taking the items together in a clinical scale instead of measuring them separately.

### Statistical Analyses

#### Model development and posttesting

Most previous studies have examined treatment progress and changes in risk factors by using repeated measures (De Jonge et al., 2009; Van der Veeken et al., 2018). In this study, latent growth curve modeling (LGCM) is used to model changes in risk factors represented by scores on the clinical risk scale between the time of the judicial psychiatric assessment until the moment of unconditional release. The sum score of the 11 items represented the clinical scale at each time point. As previously stated, the items used are scored on a 5-point scale, ranging from 0, *a very low risk at recidivism*, to 4, *a very high risk of recidivism*, meaning that the minimal score of the clinical scale is 0 and the maximum score of the clinical scale is 44. All analyses are performed via the Mplus statistical processing program version 7 (Muthén & Muthén, 2007). LGCM is a flexible statistical technique for modeling change of risk factors over time. In this research, analyses take place in three major phases, namely the unconditional model phase, the conditional model phase, and a posttesting. First, our unconditional model was tested without predictors. In this model, we examined whether the severity of the clinical scale consisting of clinical risk factors differed from each other at five time points (KPJ, KIN, KOV, KPV, and KOO^1^).

Second, and because of the heterogeneity of the forensic population under study, a conditional model is defined by adding predictors to the unconditional model. Adding predictors allow us to identify variables that predict assignment to a latent trajectory, and to test the stability of the unconditional model (Muthen, 2004). The following predictors were added to the model separately: first-time offenders compared with recidivists, age groups, and treatment duration (Jung & Wickrama, 2008). Third, bivariate postanalyses were computed. Chi-square differences tests were conducted to investigate whether the slope coefficients found for the different groups were significantly different from each other. Finally, *t* tests and analyses of variance (ANOVAs) were performed to compare the scores on the clinical scale for the different groups at the five time points. Mplus offers several fit indices to investigate the fit between the expected and observed models. Hu and Bentler (1999) use a cut-off value close to .95 for the comparative fit index (CFI) and close to .95 for the Tucker–Lewis index (TLI). For more detailed information about fit indices, see Hu and Bentler (1999).

## Results

### Descriptives

Descriptive information about the sample is found in Tables 1 and 2.

**Table 1.** Descriptive Statistics.

Variable	%	*M*	*SD*
Gender (*N* = 347)			
Males	91.4		
Females	8.6		
First-time offenders versus recidivists (*N* = 317)			
First-time offenders	11.0		
Recidivists	89.0		
Age (*N* = 317) (years)		31.86	8.72
Less than 25	26.5		
25–44years	64.7		
More than 45	8.8		
Duration (*N* = 179) (years)		70.84	16.54
Less than 5	26.3		
5–7	55.9		
More than 8	17.8		

*Note.* Age is measured in years; treatment duration is measured in months.

**Table 2.** Descriptives Statistics and Cohen’s *d* of the clinical scale on the five measurement points.

	*N*	*M*	*SD*	Cohen’s *d*
KPJ	341	20.34	6.68	
KIN	288	18.97	8.76	.19
KOV	303	14.80	7.08	.57
KPV	209	12.63	7.51	.35
KOO	346	8.61	7.44	.42

### Slope of the Clinical Scale

**Figure 1.**
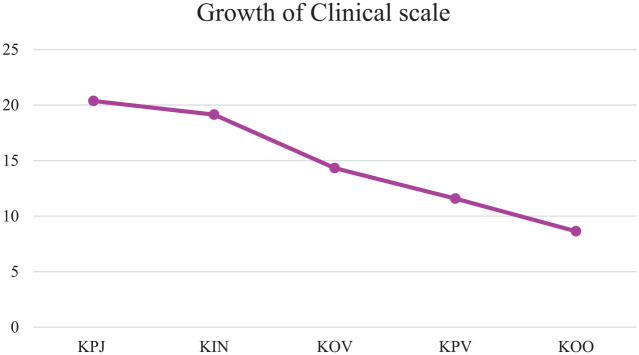
Growth trajectory of the clinical scale.

Examining the linear slope of the clinical scale at five time points resulted in an adequate model fit, which is consistent with the empirical data, CFI = .91, TLI = .91 (Hu & Bentler, 1999). The clinical scale score decreased significantly from judicial assessment until unconditional release, β = −3.28, *SE* = .15, *p* < .001. This result is presented in Figure 1. The variance around the slope was statistically significant, β = 1.45, *SE* = .39, *p* < .001, meaning that the current model does not sufficiently model individual change in clinical symptoms over time. Three subsequent analyses were performed separately and independently of each other to see whether being a first-time offender or a recidivist, age and treatment duration could contribute to the explanation of different rates of the clinical scale.

### First-Time Offenders Versus Recidivists

**Figure 2.**
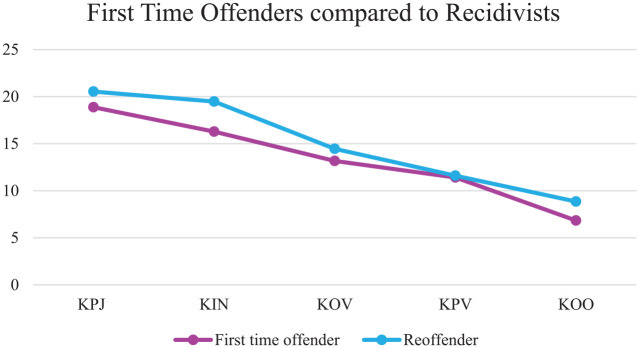
Growth trajectories of the clinical scale for first-time offenders compared with recidivists.

Examining the linear slope of the clinical scale for first-time offenders and recidivists resulted in a model that still fitted the data well, CFI = .90, TLI = .90 (Hu & Bentler, 1999). In both the first-time offender group (β = −3.443, *p* < .001) and the group of recidivists (β = −3.258, *p* ≤ .001), the slope declined significantly between the time of judicial assessment until unconditional release. To examine whether both slopes differed significantly, a chi-square difference test was performed, comparing a model with varying slopes for the two groups to a model with the same slopes. Results showed that the slope of the clinical scale did not differ significantly between first-time offenders and recidivists, χ^2^(1, *N =* 317) *=* .188, *p =* .665, indicating no difference in the growth rate of the clinical scale between the two groups. An independent samples *t*-test showed no significant differences between the groups at any of the five time points. A visual representation of the slopes can be found in Figure 2.

### Age

**Figure 3.**
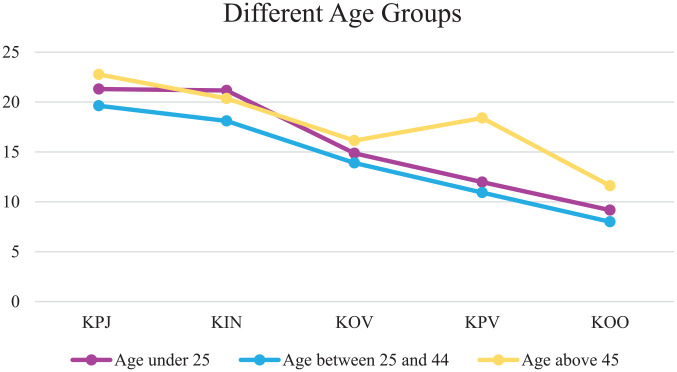
Growth trajectories of the clinical scale for the three age groups.

Examining the linear slopes of the clinical scale comparing three age groups (less than 25 years, between 25 and 45 years, and 45 years and older), resulted in a model that still fitted the data well, CFI = .90, TLI = .90 (Hu & Bentler, 1999). In all three age groups (less than 25 years: β = −3.592, *p* ≤ .001; between 25 and 45 years: β = −3.176, *p* ≤ .001; 45 years and older: β = −3.064, *p* ≤ .001), the slope declined significantly between the time of judicial assessment until unconditional release. To examine whether the slopes differed significantly between the three groups, a chi-square difference test was performed. Results showed that the slope of the clinical scale did not differ significantly between the three different age groups, χ^2^(2, *N =* 317) *=* 1.724, *p =* .422. A visual representation of the model is depicted in Figure 3.

An ANOVA was performed to compare the score on the clinical scale at the five time points for the three age groups and to investigate whether there was a significant difference between the groups. Results can be found in Tables 3 and 4. There was a significant effect of age at three of the five time points, excluding the first and third measurement point. For the second time point, post hoc comparisons using the Bonferroni correction indicated that the mean scale score 12 months after admission into a FPC differed significantly between the age group of less than 25 years (*M* = 21.16) and the age group of between 25 and 44 years (*M* = 18.10). Regarding the time of conditional release, the fourth time point, post hoc comparisons showed a significant difference for the youngest group (*M =* 11.97), compared with the oldest group (*M =* 18.40). The oldest group also differed significantly from the middle age group (*M =* 10.92). Finally, post hoc tests for the clinical scale scores at the fifth time point, the time of unconditional release, the middle age group (*M* = 7.99) differed significantly from the oldest age group (*M* = 11.61).

**Table 3.** Summary of ANOVA for the Different Age Groups.

Time point	Sum of squares	*df*	Mean square	*F*	*p*
KPJ					
Between groups	342.283	2	171.143	3.857	.022
Within groups	13,710.017	309	44.369		
Total	14,052.300	311			
KIN					
Between groups	508.314	2	254.157	3.335	.037
Within groups	19,815.223	260	76.212		
Total	20,323.538	262			
KOV					
Between groups	126.922	2	63.461	1.254	.287
Within groups	13,866.641	274	50.608		
Total	13,993.562	276			
KPV					
Between groups	529.172	2	264.586	4.849	.009
Within groups	9,985.957	183	54.568		
Total	10,515.129	185			
KOO					
Between groups	354.648	2	177.324	3.189	.043
Within groups	17,401.792	313	55.597		
Total	17,756.440	315			

**Table 4.** Bonferroni Comparison for the Three Age Groups.

Time point	Comparisons	Mean weight difference (kg)	*SE*	95% CI	
				Lower bound	Upper bound
KPJ	Less than 25 years versus 25–44 years	1.669	0.866	−0.416	3.754
	Less than 25 years versus more than 45 years	−1.463	1.454	−4.962	2.035
	25–44 years versus more than 45 years	−3.133	1.344	−6.368	0.103
KIN	Less than 25 years versus 25–44 years	3.058*	1.230	0.095	6.021
	Less than 25 years versus more than 45 years	0.810	2.091	−4.228	5.848
	25–44 years versus more than 45 years	−2.248	1.941	−6.924	2.429
KOV	Less than 25 years versus 25–44 years	0.969	0.994	−3.363	1.425
	Less than 25 years versus more than 45 years	−1.273	1.736	−5.455	2.908
	25–44 years versus more than 45 years	−2.242	1.605	−6.108	1.623
KPV	Less than 25 years versus 25–44 years	1.048	1.276	−2.036	4.132
	Less than 25 years versus more than 45 years	−6.433*	2.583	−12.673	−.193
	25–44 years versus more than 45 years	−7.482*	2.424	−13.337	−1.626
KOO	Less than 25 years versus 25–44 years	1.171	0.967	−1.156	3.498
	Less than 25 years versus more than 45 years	−2.442	1.627	−6.358	1.475
	25–44 years versus more than 45 years	−3.613*	1.503	−7.230	0.004

*Note.* CI = confidence interval.

* *p* < .05.

Taken together, the decline of clinical risk factors was not found to differ between the three age groups. However, significant group differences were found in three of the five time points. There was a significant difference at the time patients were admitted to the FPC for patients younger than 25 years compared with patients between 25 and 44 years. The clinical scale also differed significantly on the fourth time point, the time of conditional release, for the oldest age groups compared with the other two groups. Finally, the middle age group differed significantly from the oldest age group on the fifth time point, the time of unconditional release.

### Treatment Duration

**Figure 4.**
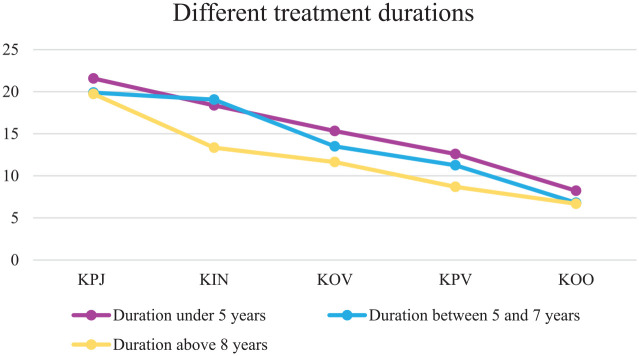
Growth trajectories of the clinical scale for the three treatment duration groups.

Examining the linear slopes of the clinical scale comparing the different treatment duration groups resulted in an acceptable model fit, CFI = .89, TLI =.89 (Hu & Bentler, 1999). In all three treatment duration groups (less than 5 years: β = −3.296, *p* ≤ .001; between 5 and 7 years: β = −3.404, *p* ≤ .001; longer than 8 years: β = −2.446, *p* ≤ .001), the slope declined significantly between the time of judicial assessment until unconditional release. To examine whether the slopes differed significantly between the three groups, a chi-square difference test was performed. Results showed that the slope of the clinical scale did not differ significantly between the three different treatment duration groups, χ^2^(2, *N =* 179) *=* 3.208, *p =* .201. A visual representation of the model is depicted in Figure 4.

An ANOVA was performed to compare the clinical scale at the five time points for the three treatment duration groups. Results can be found in Tables 5 and 6. There was a significant effect of treatment duration at the second time point. Post hoc comparisons using the Bonferroni correction indicated that the mean score on the second time point, the score after the first 12 months of stay in the FPCs, was significantly different for the duration group of between 5 and 7 years (*M* = 19.05), compared with the duration group of more than 8 years (*M* = 13.34). Regarding the time of conditional release, the fourth time point, post hoc comparisons also showed a significant difference between the duration group of less than 5 years (*M* = 12.591) compared with the duration group of more than 8 years (*M* = 8.68). This means that those receiving longer treatment showed a significantly higher score on the clinical scale compared with the group that received shorter treatment on the second and fourth time point.

**Table 5.** Summary of ANOVA for the Different Treatment Duration Groups.

Time point	Sum of squares	*df*	Mean square	*F*	*p*
KPJ					
Between groups	97.947	2	48.974	1.104	.334
Within groups	7,588.572	171	44.378		
Total	7,686.519	173			
KIN					
Between groups	639.666	2	319.833	4.512	.013
Within groups	10,349.237	146	70.885		
Total	10,988.903	148			
KOV					
Between groups	246.739	2	123.369	2.449	.090
Within groups	8,110.413	161	50.375		
Total	8,357.152	163			
KPV					
Between groups	283.078	2	141.539	2.951	.055
Within groups	8,104.577	169	47.956		
Total	8,357.656	171			
KOO					
Between groups	74.012	2	37.006	1.023	.362
Within groups	6,330.310	175	36.173		
Total	6,404.322	177			

*Note.* ANOVA = analysis of variance.

**Table 6.** Bonferroni Comparison for the Three Treatment Duration Groups.

Time point	Comparisons	Mean weight difference (kg)	*SE*	95% CI	
				Lower bound	Upper bound
KPJ	Less than 5 years versus 5–7 years	1.681	1.209	−1.242	4.604
	Less than 5 years versus more than 8 years	1.841	1.548	−1.901	5.583
	5–7 years versus more than 8 years	0.160	1.356	−3.119	3.439
KIN	Less than 5 years versus 5–7 years	−0.688	1.607	−4.580	3.205
	Less than 5 years versus more than 8 years	5.028	2.136	−.146	10.202
	5–7 years versus more than 8 years	5.716*	1.921	1.064	10.368
KOV	Less than 5 years versus 5–7 years	1.836	1.296	−1.300	4.970
	Less than 5 years versus more than 8 years	3.694	1.690	−.395	7.783
	5–7 years versus more than 8 years	1.859	1.516	−1.808	5.525
KPV	Less than 5 years versus 5–7 years	1.332	1.251	−1.693	4.357
	Less than 5 years versus more than 8 years	3.907*	1.616	−.002	7.815
	5–7 years versus more than 8 years	2.575	1.431	−.884	6.034
KOO	Less than 5 years versus 5–7 years	1.433	1.065	−1.142	4.008
	Less than 5 years versus more than 8 years	1.543	1.378	−1.789	4.875
	5–7 years versus more than 8 years	0.111	1.223	−2.846	3.067

* *p* < .05.

Taken together no significant differences were found in the growth rate of the clinical scale for the three duration groups. However, significant group differences were found at the second and fourth time point. On the time people were admitted to the FPC, patients receiving treatment for more than 8 years scored significantly lower on the clinical scale compared with the group who received treatment between 5 and 7 years. On the time patients were conditionally released, the group received treatment for more than 8 years scored lower on the clinical scale compared with the grout that received treatment less than 5 years.

## Discussion

The main goal of this study was to assess the changeability of clinical risk factors presented in a clinical scale score over time for all male forensic psychiatric patients with a TBS order, who were unconditionally released between 2004 and 2008 in the Netherlands. LGCM was performed using the Mplus statistical processing program version 7 (Muthén & Muthén, 2007). Statistical analyses were first computed for the unconditional model, then, a conditional model with three predictors (first time offenders vs. recidivists, the age of the patients and length of stay or treatment) was tested and finally, bivariate postanalyses were computed. The period of the investigation ran from the moment of juridical psychiatric assessment up to and including the moment of unconditional release. We reported on risk scale level and not on risk factor level, as we wanted to receive insight into the long-term trajectories. First, our unconditional model was tested without predictors. As we expected in our hypothesis, the latent growth curve showed a significant decrease in the severity score of the clinical scale during treatment in FPCs from the time of judicial psychiatric assessment until the time of unconditional release. This finding was in accordance with previous results found in studies assessing treatment progress (De Jonge et al., 2009). Also studies investigating short-term changes of dynamic risk factors in forensic psychiatric patients showed a decrease of problematic behavior (impulsivity, hostility, posttraumatic stress disorder [PTSD], and responsibility for the crime committed), and an increase protective and resocialization behavior based on the central eight factors, which are directly related to (violent) reoffending (Kunst et al., 2010; Schuringa et al., 2014; Van der Veeken et al., 2018).

Concerning our conditional model that was tested separately with three predictors, first-time offenders compared with recidivists at the time of the index crime showed no significant group differences in the slopes, and in any of the five time points. The decrease of clinical risk factors does not differ between recidivists and first-time offenders. However, future research must show whether forensic subpopulations, such as sex offenders, violent offender and offenders with an intellectual disability, differ in progress and decrease in size of clinical risk factors. For example, research shows that recidivists have a greater prevalence of psychiatric disorders and that the association between psychiatric disorders and recidivism is mediated by severe clinical risk factors, such as a lack of self-regulation (Lee & Hanson, 2016). Future research should certainly pay attention to specific patient groups to investigate the association between decrease of clinical risk factors and severity of the diagnosis.

Regarding age as a predictor, the three age groups were not found to differ in the growth rates of the clinical risk scale, but significant group differences were found at three of the five time points, namely the second, the fourth, and the fifth time point. Results showed that the oldest group of 45 years and older performed worse, especially at the later time points. We do not have immediate explanations for this finding, except for the general fact that the changeability of risk factors decreases with age. In general, older forensic psychiatric patients have not been studied much. There is, however, the general perception in the United States, Canada and the United Kingdom that recidivism rates of sex offenders decrease significantly in older age groups (Fazel et al., 2006). However, perception does not explain the variability of risk factors in the elderly and currently, we are not aware of any research that specifically addresses this issue. However, some explanations may be interesting, such as the perceived stigma in older patients (Pinfold et al., 2003; Sirey et al., 2001). In a study conducted by Sirey et al. (2001), it was investigated whether perceived stigma affected treatment progress in young and older adults with major depression. Results of this study showed that perceived stigma predicted treatment discontinuation among older patients only. Pinfold et al. (2003) also investigated the effect of stigma on older people with mental disorders and found that stigma leads to the development of negative attitudes that negatively influence the well-being of older patients.

Finally, group differences in treatment duration were found at one measurement point, namely the second, which indicates that the trajectories do not follow the exact same path for the three groups. However, in general there is a decrease in recidivism risk and there were no differences found in the slopes. We do not have immediate explanations for this finding based on previous research.

Even though the results should be interpreted with caution, the current study is of scientific and clinical relevance and offers first insights in the longitudinal pathways covered by clinical risk factors between the time of juridical forensic assessment and unconditional discharge. It also provides initial insight into differences between first-time offender versus recidivists, age and length of stay. In general, the studied forensic patients exhibit a significant decrease in their clinical symptoms on scale level. However, the growth rate differs between patients depending on their age and duration of stay, but not depending on whether they are first-time offenders or recidivists, indicating the effect of individual differences. However, as model fit of the unconditional model barely improved in this study after adding the predictors, these predictors showed not to be best indicators of these individual differences. This means that, to be able to provide the best treatment to every single patient, these individual differences should be looked into more carefully.

## Limitations and Directions for Future Research

Strengths of the current study encompass first the sample that is used in this study. This study was conducted in a nationwide sample, and a cohort is followed for multiple years. This is the first ever study, to our knowledge, that used such a nationwide sample that was followed for a number of years. The second strength of this study includes the use of the latent growth analysis, which is a new approach in forensic psychiatry. The use of this technique makes it possible to accurately assess treatment progress for all forensic patients and subsamples of patients related to criminal history, age and duration of stay in a forensic center. Another strength is the use of the HKT-R, as this risk assessment instrument has been validated in multiple studies and in different countries (Bogaerts et al., 2018; Spreen et al., 2013). This study provides a unique contribution to the field of forensic psychiatry and provides first insights in changes of severity on risk factors over time during treatment in FPCs, as results showed that the clinical risk factors decrease during treatment, and there appear to be differences between various patient groups.

Despite its strengths, this study also had some limitations that need to be addressed in future studies. A limitation is related to the sample size, because from the original sample of 317, only 179 patients could be included in the analysis to investigate the effect of treatment duration. Information about treatment duration or duration of stay was missing from 148 patients. This is problematic because of almost one in three patients, the FPCs have no information about the length of their stay. In addition, certain age groups and certain treatment duration groups contain a small number of patients, yielding power issues in the chi-square difference tests (Schermelleh-Engel et al., 2003).

Another limitation in this study is that we did not include other variables that might have an influence on progression of the clinical risk scale. Results showed significant variance in the slope of the clinical risk scale, indicating individual differences in the growth rate of these symptoms. However, the three predictors included in this study, being a first-time offender or a recidivist, age, and treatment duration did not show significant differences in the growth rate. Because of the small sample size of female patients, they were excluded from the analyses. Future research should assess multiple predictors that might explain be important in treatment progression, such as potential gender differences that may exist in the treatment progress of patients. In addition, each predictor was assessed separately, whereas there may be interaction effects. There appear to be individual differences in the development of the clinical risk factors, and it is of great importance that future research focuses on these individual differences, to be able to treat every patient better.

A third limitation was the assessment of the clinical items as a scale. The 11 items were taken together into one clinical risk scale and not separately, which was impossible due to a lack of power. However, it is possible that there are different growth rates on each of the clinical risk items. This study has shown that recidivism risk in general decreases during treatment, but it does not yet provide insights into which independent risk factor shows especially a decrease. It would be interesting for future research to assess treatment progress on every risk factor separately. Behavioral treatment focuses on the reduction of risk factors, and the reinforcement of protective factors, to reduce the risk of recidivism. Assessing the growth trajectories of each individual risk factor can give insights into the change of the risk and protective factors separately, indicating the areas in which treatment could still be improved, to fit the needs of every patient. A fourth limitation relates to a comparison group. We only investigated forensic patients who left the institution. These patients are characterized by a decrease in risk factors and an increase in protective factors. Future research could also include patients who are not allowed to leave the institution and for whom no reduction of risk factors and increase of protective factors can be observed.

## Conclusion

The current study demonstrates a significant decrease in the clinical risk scale consisting of risk factors in forensic patients treated in FPCs, indicating a significant decrease of risk of recidivism. Three predictors were added and differences were found at various time points, which indicates that the trajectories did not follow the exact same path for the different groups. However, in general, there is a decrease in severity of risk factors and there were no differences found in the slopes. The current study found some effects of age and treatment duration, but future research should look more closely into various individual differences that can affect treatment progress. When these individual differences can be considered in the treatment process, it can lead to better outcomes for both the patient and society.

## Notes

1. KPJ is the first time point that refers to the time of judicial psychiatric assessment; KIN is the second time point that refers to the score after the first 12 months of stay in the FPCs; KOV is the third time point related to the score before the first unguided leave; KPV is the fourth time point and is related to the score before conditional leave; KOO is the fifth time point, which refers to the score before unconditional release.
